# Engineering of monobody conjugates for human EphA2-specific optical imaging

**DOI:** 10.1371/journal.pone.0180786

**Published:** 2017-07-07

**Authors:** Min-A Kim, Hee Seung Yoon, Seung-Hwan Park, Dong-Yeon Kim, Ayoung Pyo, Hyeon Sik Kim, Jung-Joon Min, Yeongjin Hong

**Affiliations:** 1 Department of Microbiology, Chonnam National University Medical School, Gwangju, Republic of Korea; 2 Department of Molecular Medicine (BK21Plus), Chonnam National University Medical School, Gwangju, Republic of Korea; 3 Department of Nuclear Medicine, Chonnam National University Medical School, Gwangju, Republic of Korea; 4 Biological Resource Center, Korea Research Institute of Bioscience & Biotechnology (KRIBB), Jeongeup, Republic of Korea; Rutgers University, UNITED STATES

## Abstract

In a previous study, we developed an E1 monobody specific for the tumor biomarker hEphA2 [PLoS ONE (2015) 10(7): e0132976]. E1 showed potential as a molecular probe for *in vitro* and *in vivo* targeting of cancers overexpressing hEphA2. In the present study, we constructed expression vectors for E1 conjugated to optical reporters such as *Renilla* luciferase variant 8 (Rluc8) or enhanced green fluorescent protein (EGFP) and purified such recombinant proteins by affinity chromatography in *E*. *coli*. E1-Rluc8 and E1-EGFP specifically bound to hEphA2 in human prostate cancer PC3 cells but not in human cervical cancer HeLa cells, which express hEphA2 at high and low levels, respectively. These recombinant proteins maintained >40% activity in mouse serum at 24 h. *In vivo* optical imaging for 24 h did not detect E1-EGFP signals, whereas E1-Rluc8 showed tumor-specific luminescence signals in PC3 but not in HeLa xenograft mice. E1-Rluc8 signals were detected at 4 h, peaked at 12 h, and were undetectable at 24 h. These results suggest the potential of E1-Rluc8 as an EphA2-specific optical imaging agent.

## Introduction

Various scaffold proteins have been developed and used for diagnostic and therapeutic purposes in many human diseases [1]. Among these, monobodies are small scaffold proteins (molecular weight, ~10 kDa) constructed using the human fibronectin type III (Fn3) domain [2, 3] with specificities against a wide range of target molecules conferred by variations in the amino acid sequences of their three loop regions [4, 5].

Eph receptors belong to the membrane-bound tyrosine kinase family of receptors and bind to ephrin ligands on counterpart cells [6]. Their structures comprise an N-terminal extracellular region including an ephrin binding motif, a transmembrane region, and a C-terminal cytoplasmic region including a tyrosine kinase domain [7]. Binding of Eph receptors to ephrins affects a wide range of biological functions, such as cell-cell communication, cell attachment to the extracellular matrix, cell shape, and motility. Eph receptors are tumor biomarkers; an elevation in the expression of Eph receptors and ephrins correlates with a poor prognosis in various cancers [6, 8].

EphA2 is a member of the Eph receptor family. Human EphA2 gene (*hEphA2*) is located on chromosome 1 and encodes a protein with a molecular weight of 130 kD [9]. hEphA2 shows 90% amino acid sequence homology to mouse EphA2 but 25–35% homology with other Eph receptors [10, 11]. hEphA2 binds to ephrin A1 and A5, and the crystal structures of the extracellular domain of EphA2 bound to its ligands were recently determined [12–14]. The binding of EphA2 to ephrin A1 induces forward and reverse signaling in corresponding cells [15]. Cell-cell contact is lost during tumorigenesis, resulting in the disruption of normal EphA2-ephrin A1 signaling. EphA2 overexpression on tumor cells is associated with modulation of signal transduction pathways involved in cytoskeletal modulation, cell adhesion, migration, metastasis, proliferation, and angiogenesis. hEphA2 is highly expressed in various cancers, including prostate, breast, colon, pancreatic, lung and melanoma [16–19]. EphA2 is expressed during tumor neovascularization [20]. Untransformed epithelial cells become tumorigenic and metastatic after hEphA2 transfection [21]. Conversely, tumor cells in which EphA2 expression is down-regulated by small interfering RNA show reduced tumorigenic activity, such as slower cell proliferation, tumor sphere growth, and cell migration [22].

A number of hEphA2-targeting agents have been developed for therapeutic and diagnostic purposes, including monoclonal antibodies [23–25], peptides [26, 27], ephrin A1 [28, 29], small molecule inhibitors [30, 31], adenovirus vectors [30], and nanoparticles [32]. In previous work from our group, a hEphA2-specific E1 monobody (<2 nM Kd) was isolated by screening a yeast surface display library [33]. E1 binds to human tumor cells and xenograft tumor tissues expressing high levels of hEphA2 on their cell surfaces.

Many reporter proteins are used for optical imaging [34, 35]. One such protein, *Renilla* luciferase, produces luminescence in the presence of substrates such as coelenterazine. A variant, RLuc8, is more stable in serum and shows increased light production [36]; thus, it has been employed as a reporter protein for luminescence imaging in xenograft mouse models [37]. See comment in PubMed Commons belowGreen fluorescent protein emits fluorescence when excited at 488 nm, and its mutant, enhanced green fluorescent protein (EGFP), shows considerably higher fluorescence and efficient folding in *E*. *coli* [38]. In the present study, we purified conjugates of E1 monobody with Rluc8 or EGFP, and investigated their hEphA2-targeting capability in hEphA2-expressing cancer cells *in vitro* and *in vivo*.

## Methods and materials

### Cell lines

PC3 (prostate cancer) and HeLa (cervical cancer) cells were obtained from the American Type Culture Collection and grown in RPMI 1640 and high-glucose Dulbecco’s modified Eagle’s media (DMEM) containing 10% fetal bovine serum (FBS) and 1% penicillin-streptomycin in a 5% CO_2_ incubator.

### Plasmid construction

To construct expression vectors for E1-Rluc8 and E1-EGFP proteins, the fragments with the E1 monobody gene and C-terminal 2×G4S linker were first amplified with 94oldF [33] and 94old(GC)-R (5'-CAGGCCCTGCAGAAGCTTGAATTCgctgccgccgccgccgctgccgccgccgccgctTGTTCGGTAATTAATGGAAATTGG) primers against pCT-Fn3-EphA2(E1) [33]. The amplified PCR fragments were cloned into the pGEM^®^-T Easy vector (Promega, WI) to generate pGEMT-E1-C. After Eco*R*I and *Pst*I digestion of this plasmid, the 0.3 kb fragment including the E1 gene was cloned into the same enzyme sites in pBluescript SK(+) (Stratagene, Agilent, CA) to generate pBS-E1-C. Finally, the 0.3 kb *Nhe*I and *Bam*HI fragment including the E1 gene was cloned into the same enzyme sites in the pETh vector [33] and named pETh-E1GS. The Rluc8 gene fragment was amplified with Rluc8-F3 (5'- CGATGGGAATTCGCTTCCAAGGTGTACGA) and Rluc8-R3 (5'- CAGGCCGGATCCAAGCTTCTGCTCGTTCT) primers against pBAD-Rluc8 [37]. The EGFP gene fragment was amplified with GFP-F1 (5'- CGATGGGAATTCGTGAGCAAGGGCGAGGA) and GFP-R1 (5'- CAGGCCGGATCCCTTGTACAGCTCGTCCA) primers against pcDNA3-EGFP (Addgene plasmid 13031). The amplified Rluc8 and EGFP gene fragments were digested with Eco*R*I and *Bam*HI, and then cloned into the same enzyme sites of pETh-E1GS to generate pETh-E1-Rluc8 and pETh-E1-EGFP, respectively. Both plasmids of E1-Rluc8 and E1-EGFP with the C-terminal 6×His tag were confirmed by sequencing. All transformations and plasmid purifications during cloning were performed in *E*. *coli* DH5a cultured in LB medium.

### Purification of proteins by affinity chromatography

Protein purification was performed in *E*. *coli* BL21Star(DE3) (Invitrogen, CA) transformed with bacterial expression vectors as described previously [33]. Briefly, *E*. *coli* transformants with pETh-E1-Rluc8 and pETh-E1-EGFP were cultured in 5 mL of LB medium with kanamycin (50 μg/mL) overnight. For large scale expression, 2 mL of *E*. *coli* was inoculated into 2 L of LB broth with kanamycin. Bacteria were cultured at 37°C and 250 rpm for 2–4 h, and 1 mL of 1 M isopropyl β-D-1-thiogalactopyranoside (IPTG) was added. After incubation overnight, the medium was harvested by centrifugation at 8000 rpm at 4°C. The pellet was resuspended in 30 mL of Dulbecco’s phosphate buffered saline (DPBS) and lysed by sonication for 30 min at 4°C. Supernatants were obtained by centrifugation at 10,000 rpm at 4°C and applied to a HisTrap FF column (GE Healthcare Biosciences, PA) in an AKTA FPLC system (GE Healthcare Biosciences). The flow-through was discarded. The proteins bound to the column were washed with one column volume of wash buffer (DPBS) and eluted with a gradient of 0–250 mM imidazole in DPBS.

The signals from the purified proteins were detected with the NightOWL *in vivo* imaging system (Berthold Technologies, Germany). E1-Rluc8 at 672.5 M dissolved in 50 μL of PBS in Eppendorf tubes was mixed with 50 μL of 40 μg/mL coelenterazine (Biotium, CA) for 1 min to emit luminescence. E1-EGFP fluorescence was measured at the same concentration in 100 μL of PBS.

### Western blot analysis

Total hEphA2 protein expression was measured by western blotting. Cell pellets (8 × 10^5^ cells) were mixed with SDS sample buffer and boiled for 10 min. After centrifugation, supernatants were separated by 8% SDS-PAGE and transferred onto a nitrocellulose membrane (Bio-Rad, CA). The membrane was blocked for 1 h with 5% skim milk in PBS containing 0.1% Tween-20, washed twice with PBS-T, and then incubated with rabbit anti-EphA2 (1:1000 dilution, Santa Cruz Biotechnology, CA) and horseradish peroxidase-conjugated goat anti-rabbit IgG (1:10000 dilution, Life Technology, NY) in combination. As a control, anti-β-actin (1:1000 dilution, Santa Cruz Biotechnology) was used in the same membrane. Membranes were visualized with the LAS-3000 image reader (Fuji Film, Japan) using a luminal reagent (Santa Cruz Biotechnology).

### Flow cytometry analysis

Cells were detached from culture dishes with a non-enzymatic cell dissociation solution (Invitrogen) and resuspended in PBS containing 1% bovine serum albumin (PBSA). To stain for hEphA2, cells (5 × 10^5^) were incubated with mouse anti-EphA2 antibody (1:10000 dilution, R&D systems, MN) in 100 μL of PBSA for 10 min on ice. After a simple wash with 1 mL of PBSA, the cells were incubated with Alexa 488-conjugated anti-mouse IgG antibody (1:500 dilution, Invitrogen) for 10 min on ice. To detect bound E1-Rluc8, the same number of cells was incubated with 30 nM E1-Rluc8 in 100 μL of PBSA for 10 min on ice and restained with mouse FITC-conjugated anti-6×His antibody (1:1000 dilution, Abcam, UK) in 100 μL of PBSA for 10 min on ice after a simple wash. To stain the cells with E1-EGFP, cells were incubated with 67.25 nM E1-EGFP in 100 μL of PBSA for 10 min on ice. After staining, the cells were washed with PBSA, and the mean fluorescence intensity (MFI) was measured and analyzed with a FACSCalibur flow cytometer (BD Biosciences, CA).

### Cell and tissue staining

Cells (1 × 10^4^) were cultured on cover glasses on 6-well plates for 24 h. To detect bound E1-Rluc8, cells were incubated with 25.5 nM E1-Rluc8 and rabbit anti-hEphA2 antibody (1:10000 dilution, Santa Cruz Biotechnology), in 3 mL of culture medium containing 3% FBS at 37°C for 2 h. After the plates were washed with 1% BSA in PBS-T, they were incubated in FITC-conjugated anti-6×His (1:1000 dilution) and Alexa 555-conjugated anti-rabbit IgG antibodies (1:1000 dilution) for 2 h in the dark at room temperature (RT). After washing, the cover glasses were observed with an Olympus fluorescence microscopy (Olympus, Japan). To detect bound E1-EGFP, cells were incubated with 672.5 nM E1-EGFP and rabbit anti-hEphA2 antibody and counterstained with Alexa 555-conjugated anti-rabbit IgG antibody.

PC3 and HeLa tumor tissues were obtained from subcutaneous xenograft nude mice and dissected as a section using a freezing microtome (Thermo Fisher Scientific, MA). The slides with sections were blocked with 5% BSA in PBS-T for 30 min, washed once with 1% BSA in PBS-T, and stained with E1-conjugated proteins (25.5 nM E1-Rluc8 or 672.5 nM E1-EGFP) and rabbit anti-hEphA2 antibody at RT for 2 h. After washing, the tissues were stained using the same secondary antibodies used in the cell staining method described above. Fluorescence images were acquired using a fluorescence microscope.

### Protein stability in serum

Aliquots containing 60 μg of protein in 150 μL of PBS were mixed with an equal volume of mouse serum. After incubation at 37°C for the indicated times, luminescence or fluorescence signals from each sample were measured in 96-well black plates (Thermo Fisher Scientific) with the Orion L Microplate Luminometer (Berthold Detection Systems, Pforzheim, Germany) and the Infiniti M200 laser scanner (Tecan, Switzerland). For analysis of E1-Rluc8, 10 μL of 40 μg/mL coelenterazine was added to emit luminescence before measurements.

### Optical imaging by E1-Rluc8 in tumor bearing mice

Male BALB/c athymic nu-/nu- mice (6 weeks old) were purchased from the Orient Company, Korea. Mice were subcutaneously transplanted with PC3 and HeLa cells as described previously [33]. Animal care, all experiments, and the euthanasia procedures were performed in accordance with protocols approved by Chonnam National University Animal Research Committee (Permit Number: HCRL 16–001). Anesthesia was performed using a mixture of ketamine and xylazine (200 mg/kg) for implantation or 2% isoflurane for imaging. After transplantation of cells (5 × 10^7^ cells in 100 μL of PBS), tumors were grown to a size of 100–150 mm^3^ for 3 weeks. E1-Rluc8 (6 or 60 μg) was intravenously injected via the tail vein. A bioluminescent signal was immediately measured after injection of 400 ng of coelenterazine in 100 μL of PBS. Luminescence images of the mice were obtained with the NightOWL *in vivo* imaging system at the indicated times. The photon signals were quantified after tumor area gating. Tumor tissues were collected from PC3 tumor mice at the indicated time points after E1-Rluc8 injection and stained with FITC-conjugated anti-6×His antibody to measure the remaining E1-Rluc8 in the tumors.

### Statistical analysis

Statistical analysis was performed using the two-tailed Student’s t test or two-way ANOVA. A P-value of <0.05 was considered statistically significant (P < 0.05). Data are expressed as mean values ± standard deviation.

## Results

### Purification of E1-reporter proteins

**Fig 1:**
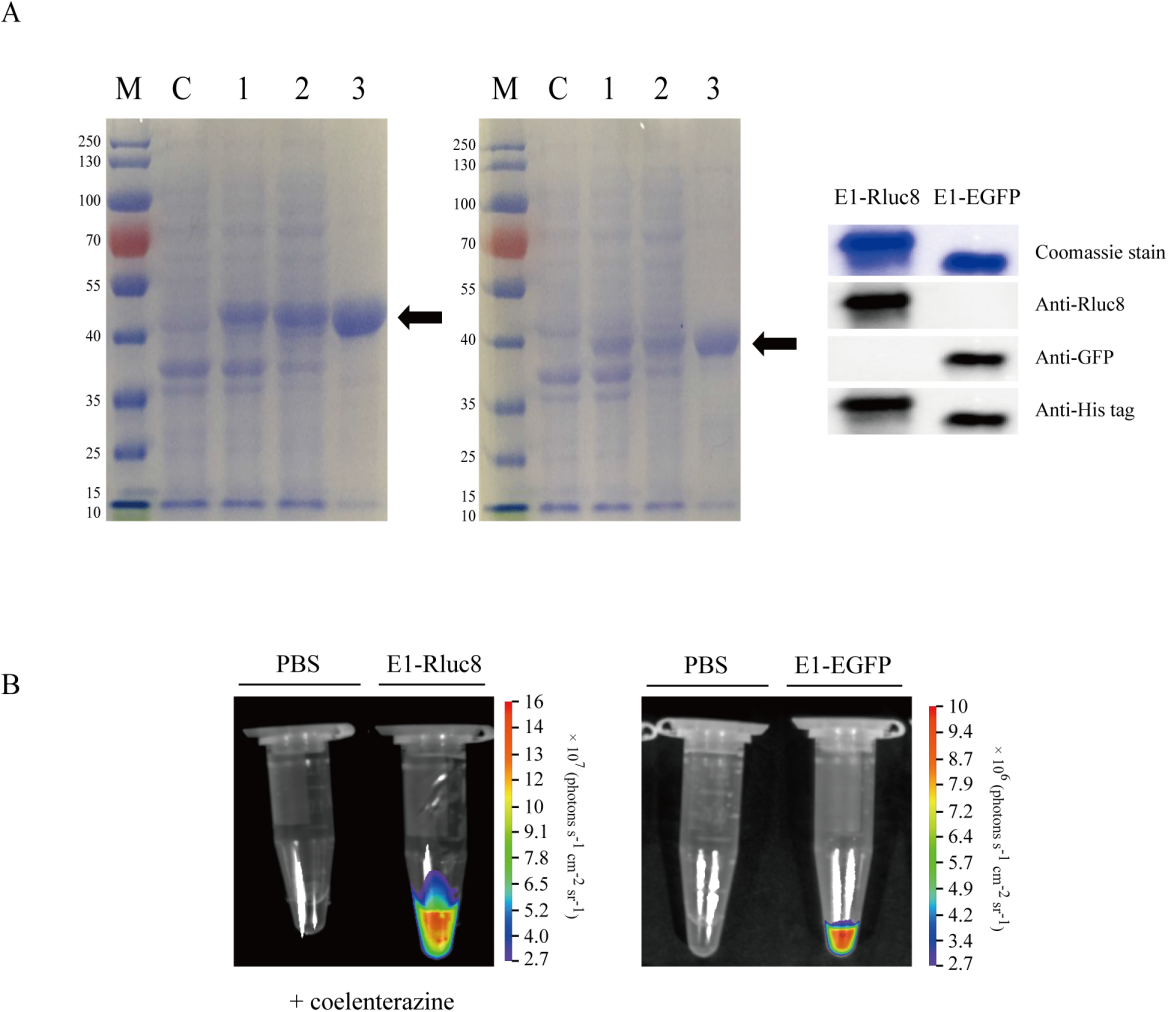
Purification and visualization of E1-reporter monobodies. (A) Expression of E1 monobodies conjugated to Rluc8 (left) and EGFP (right) in *E*. *coli*. The proteins were purified by HisTrap affinity chromatography. The purified proteins (arrows) were separated by SDS-PAGE and stained with Coomassie blue. M, protein marker; C, non-transformed bacterial lysates; 1, whole lysates from transformed bacteria (one OD_600_ equivalent); 2, supernatants from transformed bacteria; 3, purified proteins (100–200 μg). (B) Light signals from E1-Rluc8 and E1-EGFP. Purified E1-Rluc8 and PBS were mixed with coelenterazine for 1 min, and luminescence was imaged with the NightOWL *in vivo* imaging system (left). The fluorescence from E1-EGFP was directly imaged with the same system (right).

To develop the hEphA2-binding monobody conjugated to a light-emitting reporter protein, expression vectors including N-terminal E1 and C-terminal reporter genes were constructed with the pETh plasmid, yielding final recombinant proteins with a C-terminal 6×His tag. These proteins were transformed into *E*. *coli* BL21Star(DE3) and induced by IPTG, and the recombinant His-tagged E1-Rluc8 (48.3 kD) and E1-EGFP (38.9 kD) proteins were purified using affinity chromatography (Fig 1A). The purified proteins emitted a luminescent signal for E1-Rluc8 in the presence of coelenterazine or a fluorescent signal for E1-EGFP, confirming that the light-emitting reporter was functional (Fig 1B).

### *In vitro* targeting specificity of E1-reporter proteins for hEphA2

**Fig 2:**
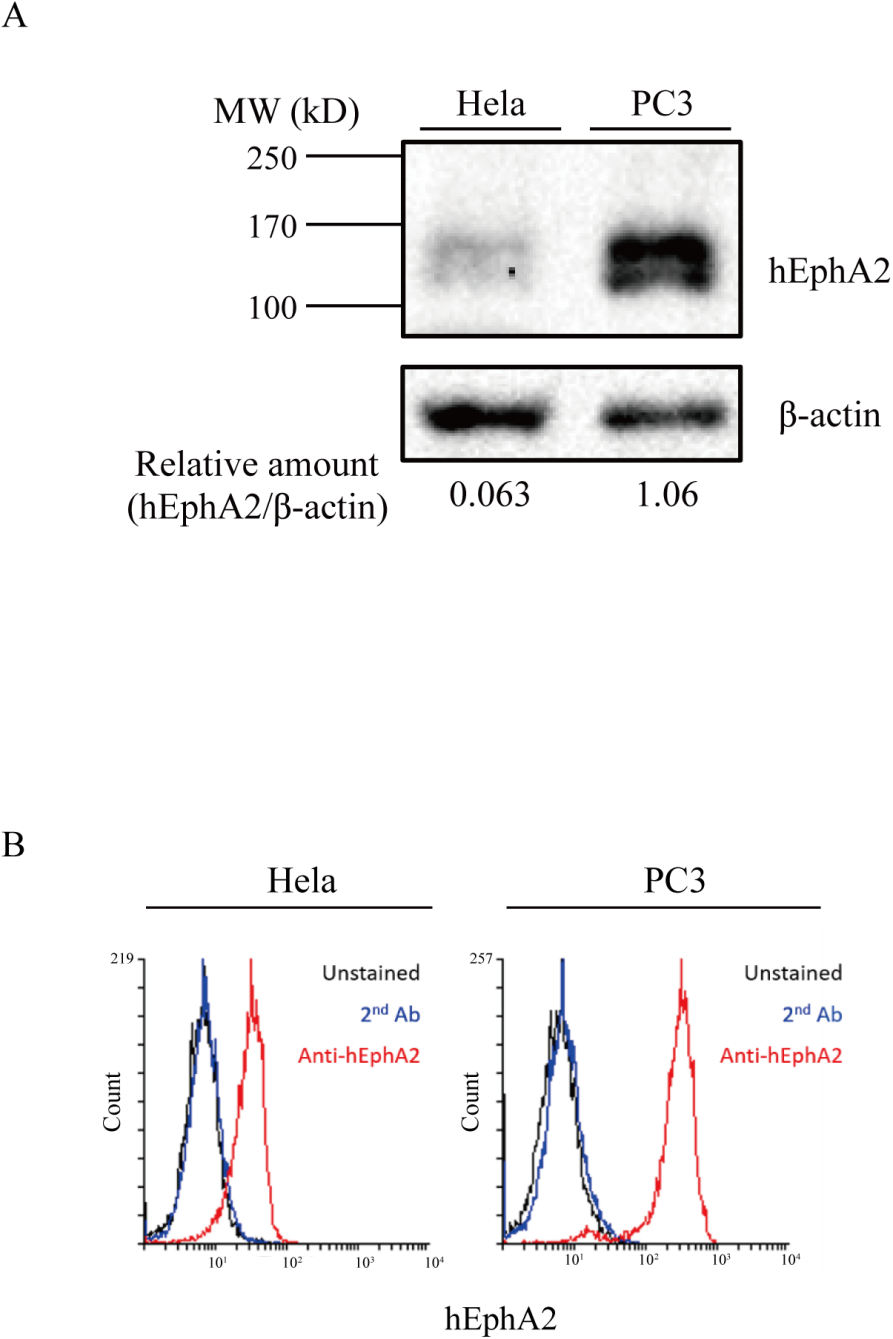
hEphA2 expression in tumor cells. (A) Western blot analysis of hEphA2. Lysates of PC3 and HeLa cells (8 × 10^5^) were separated by SDS-PAGE and transferred to nylon membranes. Membranes were stained with rabbit anti-hEphA2 or β-actin antibodies. (B) Flow cytometric analysis of cells. Cells (5 × 10^5^) were stained with mouse anti-hEphA2 and Alexa 488-conjugated anti-mouse IgG antibodies (anti-hEphA2), and fluorescence was measured by flow cytometry. Unstained cells or cells stained with Alexa 488-conjugated anti-mouse IgG antibody (2^nd^ Ab) alone were used as controls.

To examine the expression of hEphA2 on the membrane of cancer cells, two cell lines were selected, PC3 (prostate cancer) and HeLa (cervical cancer), because they express hEphA2 at high and low levels, respectively [33, 39]. hEphA2 expression levels were initially measured in both cell lines (Fig 2). Western blot analysis of whole cell extracts detected a band of approximately 130 kD in both cell lines; however, signals were faint in HeLa cells, and hEphA2 levels were 16-fold higher in PC3 cells than in HeLa cells (Fig 2A). Next, cell surface hEphA2 levels were measured by flow cytometry (Fig 2B). Staining with antibody against hEphA showed that the fluorescence intensity was 7.7-fold higher in PC3 cells than in HeLa cells, with MFI values for the anti-hEphA2 antibody and secondary antibody stained cells of 298.9 and 9.5 in PC3 and 32.2 and 8.0 in HeLa, respectively. These results indicated that hEphA2 was expressed at higher levels in PC3 cells than in HeLa cells, which was consistent with previous data [33, 39].

**Fig 3:**
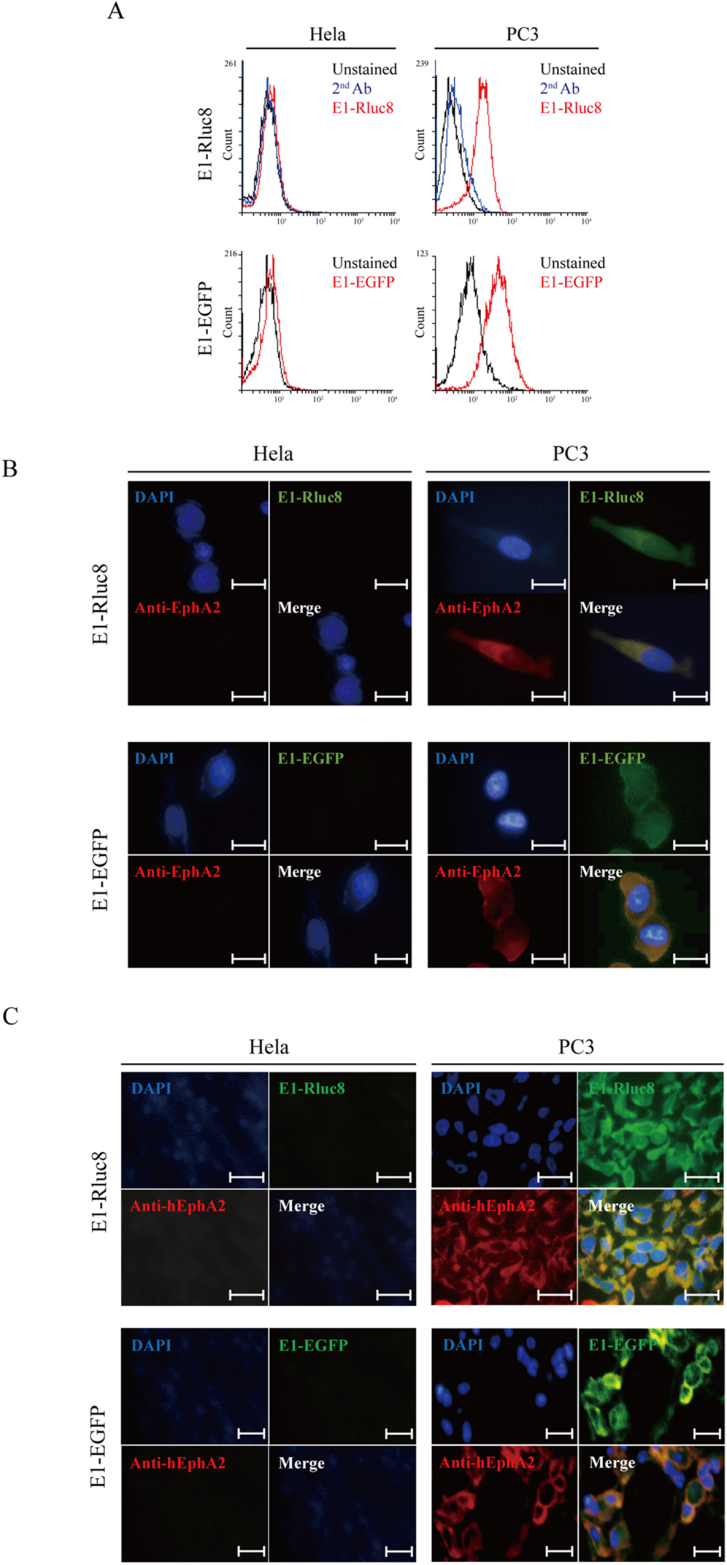
E1-reporter monobody cell binding. (A) Flow cytometric analysis of cells treated with E1-Rluc8 and E1-EGFP. PC3 and HeLa cells (5 × 10^5^) detached from culture plates were treated with E1-Rluc8 and E1-EGFP. The cells treated with E1-Rluc8 were stained with FITC-conjugated mouse anti-6×His antibody. Unstained cells or cells stained with FITC-conjugated secondary antibody (2^nd^ Ab) alone were used as controls. (B) Fluorescence microscopy images of the cells. PC3 and HeLa cells were stained with mouse anti-hEphA2 antibody and E1-Rluc8, and then detected with Alexa 555-conjugated anti-mouse IgG (red) and FITC-conjugated anti-6×His antibodies (green). Cell nuclei were stained with DAPI (blue). Scale bar, 10 μm. (C) Fluorescence microscopy images of xenograft tumor tissues. PC3 tumor tissues from transplanted nude mice were stained with the antibody combinations used in (B). Scale bar, 10 μm.

Next, we tested whether the E1-reporter proteins specifically bound to both cells. To measure the specific binding of E1-Rluc8, cells were incubated with 30 nM E1-Rluc8 and FITC-conjugated anti-6×His antibody (Fig 3A, upper panels). PC3 cells treated with E1-Rluc8 showed a 4-fold higher MFI than those treated only with secondary antibody, whereas HeLa cells did not show significant differences. The MFIs of E1-Rluc8 and secondary antibody stained cells were 18.1 and 4.4 in PC3 and 6.4 and 5.4 in HeLa, respectively. For E1-EGFP staining, cells were incubated with 67.25 nM E1-EGFP, which resulted in a similar increase in fluorescence in PC3 but not in HeLa cells. The fluorescence microscopy images obtained with the anti-hEphA2 antibody, E1-Rluc8, and E1-EGFP were similar in PC3 cells, whereas fluorescence signals were not observed in HeLa cells (Fig 3B). The MFIs of E1-EGFP stained and unstained cells were 55.2 and 11.2 in PC3 and 8.2 and 4.9 in HeLa, respectively. The fluorescence microscopy images of xenograft tumor tissue expressing either E1-Rluc8 or E1-EGFP were similar (Fig 3C). Concentrations of 25.5 nM E1-Rluc8 and 672.5 nM E1-EGFP were used for cell and tissue staining. These results demonstrated that the E1-reporter monobodies were functionally bound to hEphA2.

### *In vivo* targeting of E1-Rluc8 in a mouse tumor model

**Fig 4:**
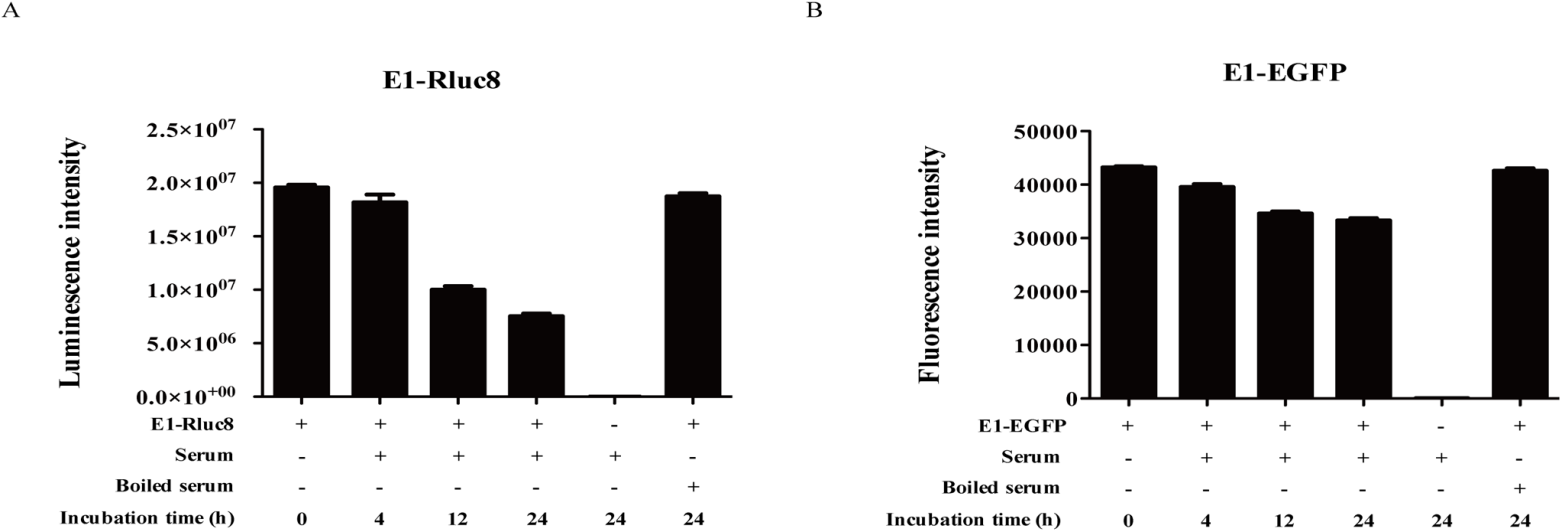
Stability of E1-reporter monobodies in serum. (A) Luciferase activity measurement of E1-Rluc8. The proteins were incubated with mouse serum for the indicated times. The light emitted from E1-Rluc8 was measured using a luminometer after treatment with coelenterazine. E1-Rluc8 incubated in boiled serum was used as a control. (B) Fluorescence activity measurement of E1-EGFP. The fluorescence emitted by E1-EGFP was measured using a fluorometer.

The stability of E1-reporter monobodies was analyzed *in vivo* by measuring protein stability in mouse serum during incubation at 37°C for 24 h (Fig 4). The luciferase activity of E1-Rluc8 decreased to approximately 50% of that of the control at 12 h and 40% at 24 h, and the fluorescence intensity of E1-EGFP decreased to 74% of that of the control at 24 h. Although E1-EGFP was more stable than E1-Rluc8 in serum, E1-EGFP showed considerably lower photon counts than E1-Rluc8, with maximum values of 1.0 × 10^7^ and 1.6 × 10^8^, respectively.

To determine whether E1-reporter monobodies targeted hEphA2-expressing tumors in xenograft mice, E1-reporter monobodies were injected into xenograft mouse models (PC3 and HeLa) via the tail vein. In PC3 tumor bearing mice injected with 60 μg E1-EGFP, fluorescence signals were not detected at 6 and 24 h (data not shown), indicating that the fluorescence generated by E1-EGFP was not sufficient for optical imaging in the *in vivo* environment.

**Fig 5:**
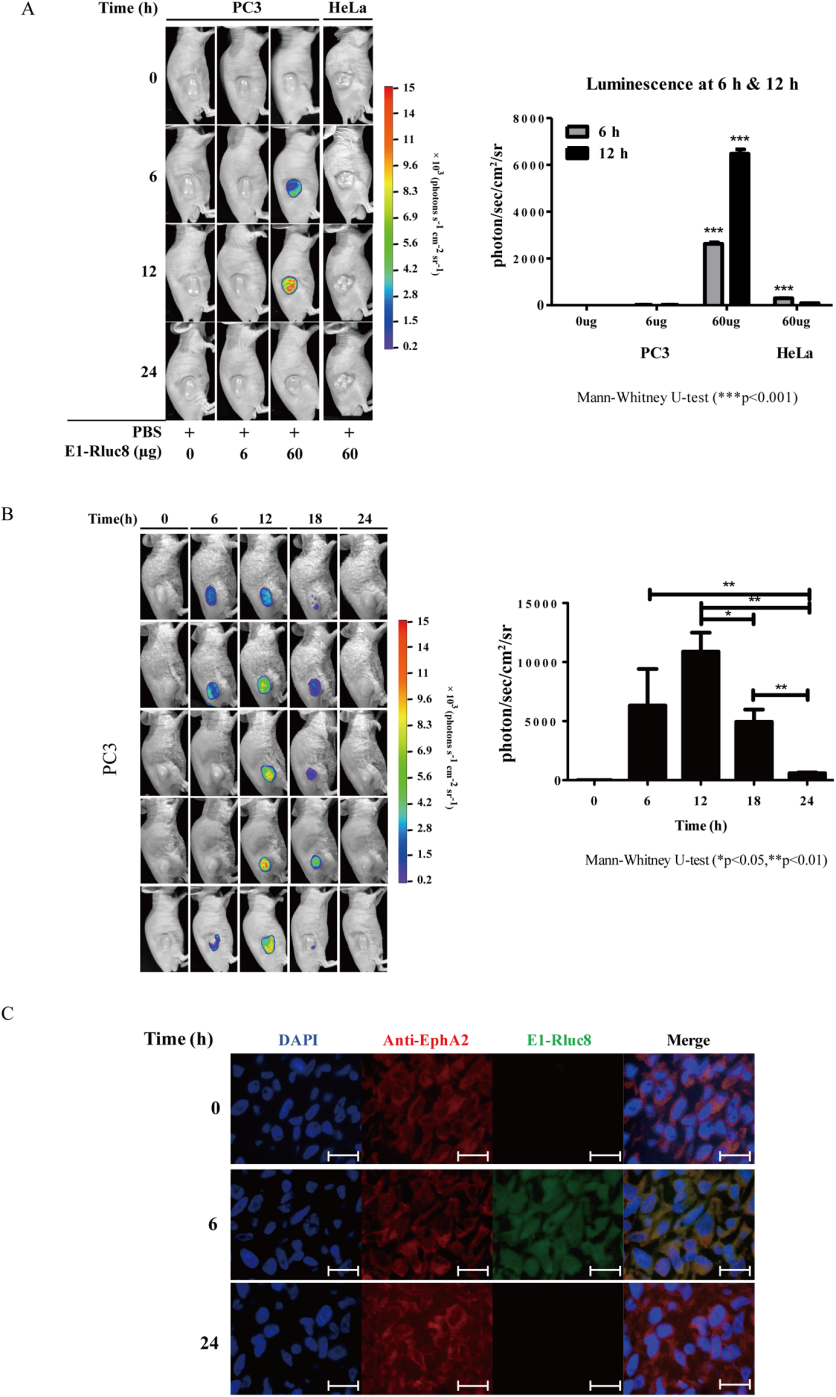
*In vivo* imaging analysis with the E1-reporter monobody in tumor xenograft mice. (A) Measurement of luminescence in mice injected with different concentrations of E1-Rluc8. PC3 and HeLa cells were transplanted into 6-week-old Balb/c nude mice via subcutaneous injection (n = 3). After tumor formation, the indicated amounts of E1-Rluc8 and coelenterazine were intravenously injected via the tail vein, and luminescence images were acquired at the indicated times with the NightOWL *in vivo* imaging system (left). The luminescence intensities measured in tumor areas at 6 h were graphed (right). (B) Luminescence maintenance in mice injected with E1-Rluc8 for 24 h. E1-Rluc8 (60 μg) and coelenterazine were intravenously injected via the tail vein into PC3 tumor mice (n = 5), and luminescence images were obtained at the indicated times (left). The luminescence intensities in tumor tissues were measured with the NightOWL *in vivo* imaging system and graphed (right). (C) Detection of the remaining E1-Rluc8 in PC3 tumor tissues. Tumor tissues were collected at the indicated times from mice injected with E1-Rluc8 and stained with FITC-conjugated anti-6×His antibodies (green). hEphA2 was stained with mouse anti-hEphA2 antibody and Alexa 555-conjugated anti-mouse IgG (red). Cell nuclei were stained with DAPI (blue). Scale bar, 10 μm.

Assessment of the optimal concentration of E1-Rluc8 to visualize targeting in tumor bearing mice showed that 60 μg of E1-Rluc8 generated a 30-fold higher luminescence signal in PC3 than in HeLa mice at 6 h. However, luminescence signals were not detected at 6 h in mice injected with 6 μg of E1-Rluc8 (Fig 5A). These results indicated that the light signals generated by 60 μg E1-Rluc8 were sufficient for *in vivo* imaging. Next, we measured the changes in luminescence signals after 60 μg E1-Rluc8 injection for 24 h (Fig 5B). Luminescence signals were detected at 6 h after E1-Rluc8 injection and peaked at 12 h. Detection of the remaining E1-Rluc8 in luminescent PC3 tumor tissues by fluorescence microscopy showed that E1-Rluc8 signals were present at 6 h, whereas they were undetectable at 0 and 24 h (Fig 5C). This indicated that the luciferase activity of E1-Rluc8 resulted in luminescence emission in PC3 mice.

## Discussion

In a previous report, we identified a hEphA2-specific E1 monobody [33]. In the present study, the E1-reporter proteins E1-Rluc8 and E1-EGFP were developed for use as optical imaging probes and purified from *E*. *coli* by affinity chromatography. The monobodies bound to both cells and xenograft tumor tissues expressing high levels of hEphA2 (Figs 3 and 4). These results indicated that both proteins possessed hEphA specificity as well as functional reporter activity. However, photon emission from E1-Rluc8 (approximately 1 × e^8^ ph/s in the maximum range) in the presence of its substrate coelenterazine was 10-fold higher than that from E1-EGFP (approximately 9 × e^6^ ph/s in the maximum range) (Fig 1). Mouse tissues emit autoluminescence and autofluorescence, and the low autoluminescence levels usually result in superior signal to background ratios for bioluminescent imaging, particularly compared with fluorescent imaging in the green to red part of the spectrum [40]. This could explain why E1-Rluc8 but not E1-EGFP produced images after injection at the same concentrations into PC3 tumor mice (Fig 5).

In our previous report, we detected fluorescent images in PC3 tumor mice with Cy5.5-labeled E1 at 5 days after injection [33]. Because it could only be detected after 5 days, this construct was not suitable for use as an imaging drug. Furthermore, fluorescence signals were detected in organs such as the kidney and liver, in addition to those in the tumor. In the present study, E1-Rluc8 signals were detected after several hours and luminescence signals were not observed in other organs (data not shown). To obtain clear images, the amount of E1-Rluc8 (60 μg) injected into mice was 10-fold higher than that of Cy5.5-E1 (6 μg).

As an *in vivo* optical imaging drug, E1-Rluc8 has several advantages. First, it is easy to prepare in an *E*. *coli* expression system. We purified an average of 50 mg protein from a 1 L culture of *E*. *coli* in the present study (data not shown). Second, its binding and enzyme activities can be assayed easily using flow cytometry and light detection by ELISA after substrate addition. In addition, the low autoluminescence levels in mouse tissues decrease background image noise. Although we did not measure the *in vivo* solubility of the probe, Rluc8 itself would be intact for 12 h because E1-Rluc8 signal were peaked at the time.

The results of the present study suggest that E1-Rluc8 is a feasible hEphA2-specific imaging agent. Modification of the E1 counterpart to a toxic protein or prodrug enzyme would enable its development as a therapeutic agent.
